# Delphi consensus of specialist radiologists on imaging criteria for gadolinium contrast use and whole-body MRI in prostate cancer

**DOI:** 10.1186/s13244-026-02388-0

**Published:** 2026-09-02

**Authors:** Eric Nduka Onwuharine, Alexander James Clark, Matthew Dimmock, Jonathan Hill

**Affiliations:** 1 School of Allied Health Professions and Pharmacy, Keele University, Keele, United Kingdom; 2 Radiology Department, University Hospitals of North Midlands NHS Trust, Stoke-on-Trent, United Kingdom; 3 School of Medicine, Keele University, Keele, United Kingdom

**Keywords:** Prostatic neoplasms, Magnetic resonance imaging, Gadolinium, Contrast media, Whole body imaging

## Abstract

**Objective:**

To achieve expert consensus on structured prostate MRI criteria for gadolinium-based contrast agent (GBCA) administration and whole-body MRI (WB-MRI) escalation in the diagnostic evaluation and staging of prostate cancer (PCa).

**Materials and methods:**

A three-round modified online Delphi survey was conducted among UK specialist prostate radiologists. Participants rated predefined clinical and MRI criteria using structured Likert agreement scales and provided free-text comments to refine decision-making. Quantitative responses were summarised descriptively. Qualitative thematic analysis informed subsequent rounds. Consensus for each decision criterion was predefined as ≥ 70% agreement. The study was conducted in accordance with CREDES (Conducting and Reporting of Delphi Studies) guidance.

**Results:**

The number of radiologists who completed Rounds 1, 2, and 3 was 21, 14, and 11, respectively. Two principal outputs were defined: (1) consensus criteria for GBCA administration; (2) consensus criteria for WB-MRI escalation. For GBCA administration, consensus supported its use for Prostate Imaging-Reporting and Data System (PI-RADS) 3 lesions (86%) and low prostate imaging quality (PI-QUAL ≤ 3; 91%), and avoidance in high-quality (PI-QUAL 4–5) images (73%). Fixed prostate-specific antigen (PSA) or PSA density thresholds did not reach consensus when considered in isolation. For WB-MRI, consensus supported escalation for T3a/T3b disease (93%), combined clinical and radiological suspicion of metastasis (91%), and high-risk profiles (PSA ≥ 20 ng/mL, Gleason ≥ 8 or ≥ T3; 86%).

**Conclusion:**

This Delphi study established expert consensus on structured MRI criteria to guide selective GBCA administration and WB‑MRI escalation, with the potential to reduce unnecessary GBCA administration, improve consistency and optimise prostate MRI workflow.

**Key Points:**

- **Question** What consensus-based criteria can guide on-table gadolinium-based contrast agent administration and whole-body MRI escalation during prostate MRI to reduce variability and unnecessary imaging?
- **Findings** Delphi consensus established imaging-based criteria integrating lesion suspicion, image quality and high-risk features to guide selective gadolinium-based contrast agent administration and whole-body MRI escalation.
- **Critical relevance statement** This article critically evaluates gadolinium-based contrast agent administration in prostate MRI and defines consensus-based standardised decision-making criteria. These criteria aim to reduce unnecessary gadolinium-based contrast agent administration and improve efficiency and consistency in staging pathways.

**Graphical Abstract:**

**Figure:**
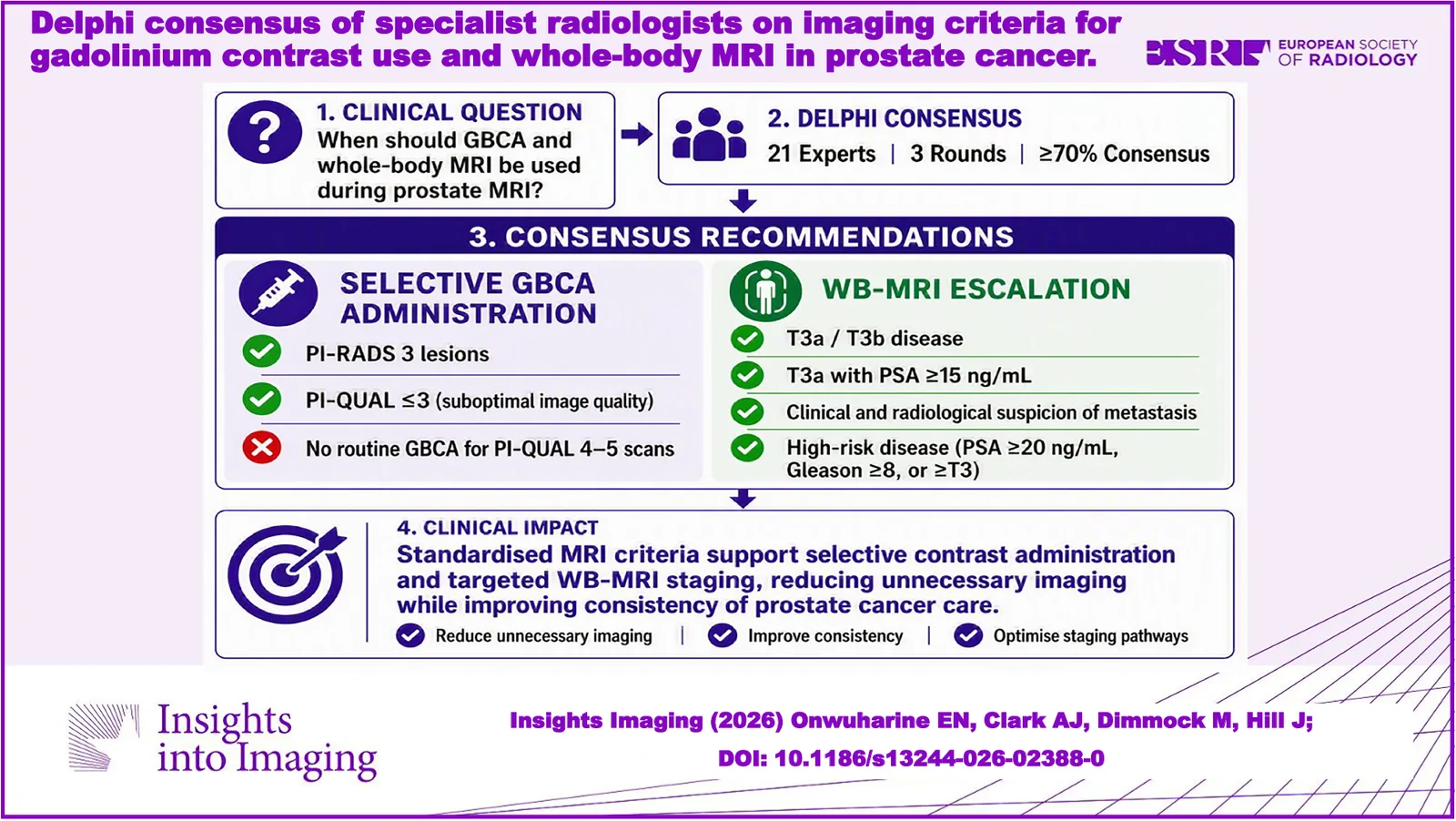

## Introduction

Multiparametric MRI (mpMRI) is the recommended first-line investigation for suspected clinically localised prostate cancer (PCa) [1]. It provides both diagnostic and prognostic information by enabling assessment of tissue functionality [2, 3]. The use of gadolinium-based contrast agent (GBCA) in mpMRI may improve diagnostic confidence in selected cases by enhancing lesion conspicuity and characterisation [4]. However, GBCA administration carries recognised risks, including adverse reactions such as nausea, vasovagal responses and chest pain [5, 6]. Concerns also persist regarding gadolinium deposition in various tissues with uncertain long-term implications [7, 8].

Biparametric MRI (bpMRI), which does not require GBCA, has demonstrated comparable accuracy to mpMRI for cancer detection in many clinical contexts [9–11]. Despite this, contrast-enhanced imaging is frequently performed by default as a precautionary strategy, particularly in cases of equivocal findings or suboptimal image quality due to artefact (e.g., hip prostheses or rectal gas) [12].

In routine practice, decisions regarding GBCA administration often occur on-table and under time pressure. Radiographers must therefore determine in real time whether additional sequences are warranted before the patient leaves the scanner. Immediate radiologist supervision is not consistently available in all centres, particularly within high-volume services operating under workforce constraints [13, 14]. Consequently, uncertainty related to PI-RADS 3 lesions, borderline image quality (e.g., PI-QUAL 3), motion artefact or susceptibility distortion may lead to precautionary GBCA administration in the absence of structured criteria. A recent update by the international PI-RADS steering committee has emphasised the importance of on-table MRI assessment to determine whether GBCA is required [15]. Establishing clear and standardised decision criteria may therefore support safer and more efficient workflow while reducing unnecessary GBCA administration.

Additionally, decisions around whole-body MRI (WB-MRI) escalation for staging present related challenges. Access to WB-MRI remains heterogeneous across NHS Trusts, with variation in scanner availability, reporting expertise and pathway configuration. While some centres have integrated WB-MRI into staging pathways for high-risk disease, others continue to rely on CT, bone scintigraphy or PSMA-PET, depending on local resources. Any consensus-based criteria addressing WB-MRI escalation must remain adaptable to differing service models rather than prescriptive of a single staging pathway.

Despite growing interest in bpMRI and WB-MRI-based staging pathways that avoid separate CT or bone scintigraphy examinations associated with exposure to ionising radiation, there are currently no structured, consensus-based criteria to support on-table decision-making. To improve both consistency and sustainability, this study therefore aimed to achieve expert consensus through a structured Delphi process to define MRI-based criteria for selective GBCA administration and on-table WB-MRI escalation during diagnostic and staging workflows. The establishment of such consensus-based criteria may provide a foundation for future development of workflow decision‑support tools; however, this study does not present or evaluate a clinical decision-support system.

## Materials and methods

### Ethical approval

Ethical approval was obtained from the University Research Ethics Committee (UREC) on 28 January 2025 (REC Project Reference: 0971). Participants were recruited through the British Society of Urogenital Radiology (BSUR), and all provided electronic informed consent. Each radiologist received an information sheet outlining study objectives, procedures, confidentiality safeguards and the voluntary nature of participation.

### Study design

A modified electronic Delphi (e-Delphi) method was used to achieve expert consensus on key aspects of clinical decision-making in prostate MRI. The Delphi approach was considered “modified” because Round 1 items were pre-generated from preparatory semi-structured interviews and a systematic review, rather than developed from a fully open-ended first round. The study was conducted electronically with controlled feedback provided between rounds. This structured, iterative approach is well-suited for synthesising expert opinion and addressing areas of clinical uncertainty [16, 17]. The study adhered to CREDES (Conducting and Reporting of Delphi Studies) recommendations [18], as detailed in Supplementary File 2.

### Questionnaire development

Preparatory work included:

- five semi-structured interviews with UK prostate MRI experts to elicit perspectives on clinical decision-making, and
- a systematic review of recent literature to support initial statement generation [19].

Findings informed the development of Round 1 items, which included closed-ended and open-ended questions to capture a broad range of viewpoints and additional decision criteria.

### Panel eligibility

Eligible participants were UK-based radiologists who:

- had ≥ 1 year of independent prostate MRI reporting experience,
- were actively practising within the NHS,
- reported ≥ 5 prostate MRI exams per week (or ≥ 150 per year), and
- were familiar with PI-RADS and contemporary prostate MRI workflows.

Both early-career and senior consultants were included to ensure diverse experiential perspectives.

### Recruitment

A purposive recruitment strategy was used. Invitations were distributed by the BSUR Research and Guidelines Officer. The exact number of recipients was not verifiable. A minimum target of 15 experts was prespecified, consistent with Delphi methodological guidance [20, 21]. Twenty-one experts participated in Round 1. Participation was voluntary and anonymous.

### Delphi survey procedures

Up to three iterative rounds were conducted using Microsoft Forms™:

- **Round 1:** Closed-ended items and open-text questions were used to capture participant perspectives.
- **Rounds 2–3:** Refined statements were presented using structured agreement formats (binary agree/disagree and Likert-type scales), informed by controlled feedback comprising aggregated group-level percentage agreement and anonymised representative quotations.

Consensus was defined a priori as ≥ 70% agreement.

**Fig. 1:**
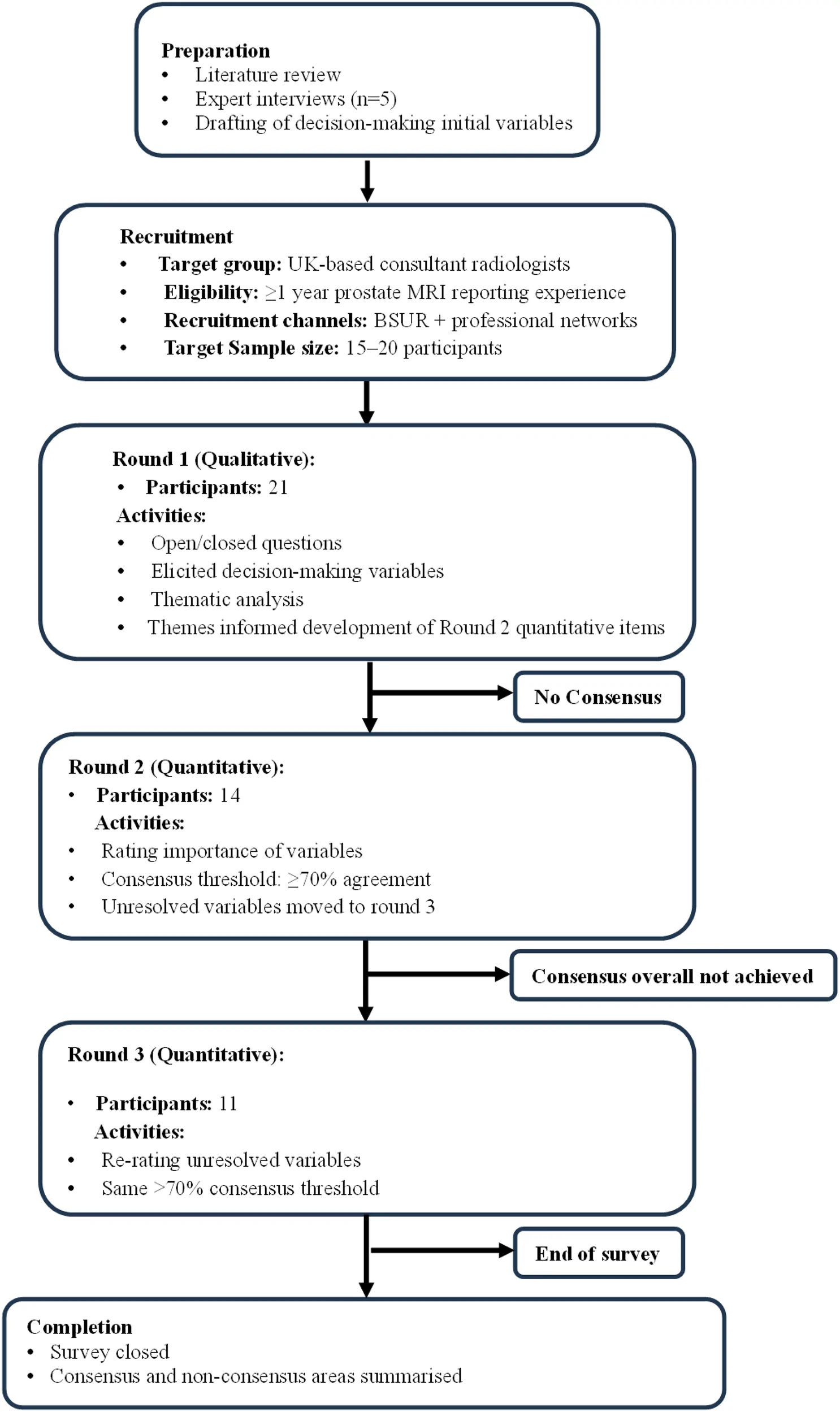
Flowchart of the e-Delphi study process

A visual summary of the Delphi process, including participant flow and rounds of data collection, is presented in Fig. 1.

### Data analysis

#### Quantitative analysis

Likert-scale and categorical responses were summarised descriptively using frequencies and proportions. Consensus was defined as ≥ 70% agreement. The distribution of responses was examined to determine how divided participants’ views were and to identify any patterns of disagreement. Items failing to reach consensus were analysed for patterns of divergence, including:

- balanced polarity (near-equal agreement/disagreement),
- clustered minority dissent, and
- context-dependent variability reflected in free-text explanations.

These analyses informed item refinement in subsequent rounds.

#### Qualitative analysis

Qualitative responses were analysed inductively. Two reviewers (E.O. and A.C.) independently coded all responses using NVivo, a qualitative data analysis software [22]. Inter-rater reliability was assessed across 54 coded units using Cohen’s kappa (κ = 0.755, 95% CI 0.617–0.866). Ninety-five per cent confidence intervals were generated using bootstrap resampling (5000 iterations), with resampling performed at the level of coded units. Discrepancies were resolved through discussion, and the resulting thematic framework informed refinement of subsequent rounds.

#### Attrition

Participation declined across Delphi rounds (21 to 14 to 11). Attrition is a recognised feature of Delphi studies and was monitored throughout.

#### Attrition bias assessment

To assess potential attrition bias, demographic and professional characteristics were compared between Round 1 participants who consented to continue to Round 2 (“continuers”, *n* = 14) and those who did not respond (“non-responders”, *n* = 7). Variables examined included professional level, primary area of expertise, years of prostate MRI reporting experience, reporting frequency and geographical region. Given the small sample size, analyses were descriptive, with percentages calculated relative to subgroup totals.

#### Transparency

The full Delphi instrument for all three rounds is included as Supplementary File 1. Figure 1 provides a visual summary of the Delphi process, participant flow and consensus development.

## Results

### Participant characteristics

Twenty-one radiologists participated in Round 1. Demographic characteristics are summarised in Table 1. Fourteen participants completed Round 2, and eleven completed Round 3.

**Table 1:** Participant demographics (Round 1, *N* = 21)

Characteristic	*N* (%)
Professional role	Consultant/attending physician—19 (90.5%) Academic title (PhD, Professor)—2 (9.5%)*
Primary area of expertise	Uroradiology—14 (66.6%) Radiology (general)—2 (9.5%) Body imaging—1 (4.8%) Gastrointestinal & urological—2 (9.5%) Prostate imaging—1 (4.8%) Cancer imaging—1 (4.8%)
Years of experience in prostate MRI reporting	< 5 years—4 (19.1%) 5–10 years—4 (19.1%) 10–20 years—10 (47.6%) > 20 years—3 (14.3%)
Prostate MRI reporting frequency	Daily—14 (66.7%) Weekly—7 (33.3%)
Geographical location (UK)	England—17 (80.9%) Scotland—1 (4.8%) Not specified—3 (14.3%)
Consent to receive future survey rounds	Yes—15 (71.4%) No—6 (28.6%)

* Some participants had dual roles (e.g., consultant plus academic title)

### Attrition comparison

Of the 21 Round‑1 participants, 14 (66.7%) continued to Round 2 and 7 (33.3%) did not respond. As shown in Table 2, the demographic and professional profiles of continuers and non-responders were broadly comparable. Consultants comprised 71.4% of continuers and 100% of non-responders. Uroradiology was the most common subspecialty in both groups (50% vs 71.4%). Years of experience were similar overall, although the 10–20‑year category was more represented among continuers (64.3% vs 28.6%). Reporting frequency showed a modest difference, with daily prostate MRI readers comprising 50.0% of continuers and 71.4% of non-responders. Geographic distribution was comparable between groups. Across all examined variables, no consistent pattern suggested systematic attrition by professional seniority, expertise, experience, reporting frequency or regional representation. This suggests that attrition between Rounds 1 and 2 was unlikely to have substantially altered the composition of the expert panel. Because Round‑1 and Round‑2 participation could be linked through explicit consent to continue, attrition between these rounds was measurable. However, subsequent rounds were anonymised, preventing identification of individuals who withdrew after Round 2 and thereby precluding further attrition analysis.

**Table 2:** Delphi attrition analysis (Round 1 to Round 2)

Characteristic	Level	Continued *n* (%)	Opted out *n* (%)	Total (*n*)
Professional level/qualification	Consultant/attending physician (fully qualified)	10 (71.4%)	7 (100.0%)	17
	Academic titles (PhD, Professor)	1 (7.1%)	0 (0.0%)	1
	Medical qualification (MD/DO, Board-certified)	3 (21.4%)	0 (0.0%)	3
Primary expertise	Uroradiology	7 (50.0%)	5 (71.4%)	12
	Radiology (general)	1 (7.1%)	1 (14.3%)	2
	Body imaging	1 (7.1%)	0 (0.0%)	1
	Gastrointestinal and urological	3 (21.4%)	0 (0.0%)	3
	Prostate imaging	1 (7.1%)	1 (14.3%)	2
	Cancer imaging	1 (7.1%)	0 (0.0%)	1
Years of prostate MRI experience	< 5 years	4 (28.6%)	2 (28.6%)	6
	5–10 years	1 (7.1%)	2 (28.6%)	3
	10–20 years	9 (64.3%)	2 (28.6%)	11
	> 20 years	0 (0.0%)	1 (14.3%)	1
Prostate MRI reporting frequency	Daily	7 (50.0%)	5 (71.4%)	12
	Weekly	7 (50.0%)	2 (28.6%)	9
Region (UK)	England	13 (92.9%)	7 (100.0%)	20
	Scotland	1 (7.1%)	0 (0.0%)	1

**Fig. 2:**
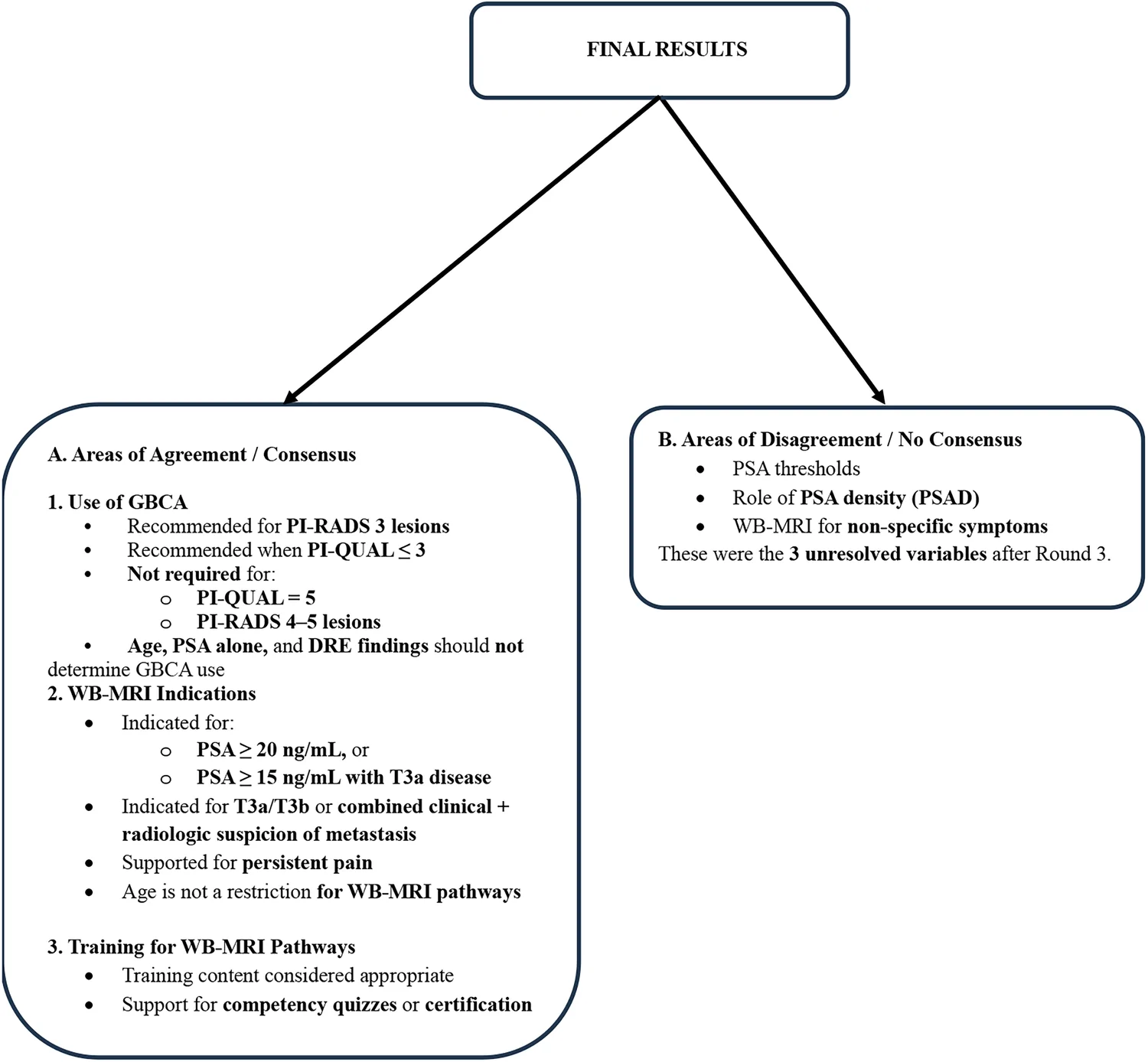
Summary of consensus and unresolved variables from the e-Delphi study

Figure 2 summarises the areas of consensus and non-consensus identified in the final round of the Delphi process.

### Round 1 results

#### GBCA administration

Participants generally considered routine GBCA administration unnecessary for an initial prostate MRI performed according to PI-RADS guidance. However, many viewed GBCA as appropriate in selected circumstances, such as equivocal PI-RADS 3 lesions or staging of biopsy-confirmed cancer.

No consensus was reached regarding the modifying role of PSA, PSA density or lesion location. Responses were distributed across agreement categories without clear clustering in the upper tiers, reflecting variability in practice.

#### WB-MRI escalation

There was broad support for WB-MRI in high-risk PCa (PSA ≥ 20 ng/mL, Gleason ≥ 8 or ≥ T3 disease). WB-MRI was generally not recommended for intermediate-risk disease unless additional clinical or imaging suspicion was present.

#### Thematic analysis

Six themes were identified from qualitative Round 1 responses:

1. Limited routine role of GBCA in initial prostate MRI.
2. Strong influence of PI-RADS 4–5 lesions on biopsy decisions.
3. Elevated PSA as a driver for WB-MRI referral.
4. MRI signs suggesting local extension or metastatic disease.
5. Integration of PSA and PI-RADS into decision-support frameworks.
6. Need for structured, stepwise decision-making pathways.

These themes informed refinement of Round 2 survey items. Representative quotations are presented in Table 3.

**Table 3:** Summary of thematic analysis from Round 1

Theme	Representative quote
Clinical and imaging decision factors	
Standard GBCA usage unless biopsy is indicated.	“I consider contrast in prostate MRI to be standard, that is the decision is when it is NOT needed”.
High PI-RADS scores (4–5) drive biopsy decisions.	“PI-RADS 4 and 5 lesions will require biopsy and contrast can be avoided”.
Elevated PSA (> 20 ng/mL) suggests consideration of WB-MRI.	“Strong impression there is prostate cancer (PI-RADS 4 and 5) with a high PSA (over 20) …”
MRI signs of local extension or metastases.	“Evidence of disease outside of the prostate on the MRI (local extension, nodal or bone disease …)”
Structured decision-making considerations	
Integration of PSA and PI-RADS in decision rules.	“PI-RADS 4 and 5; PSA only relevant as part of Likert score”.
Support for stepwise, structured decision-making.	“Stepwise approach … giving confidence to radiographers that have made all considerations”.

### Round 2 results

Fourteen participants completed Round 2.

#### Consensus achieved

- PI‑RADS 3 lesions require GBCA administration (≥ 70% agreement, with clustering in higher agreement categories).
- PI‑QUAL ≤ 3 should prompt consideration of GBCA.
- PSA ≥ 20 ng/mL is an appropriate trigger for WB‑MRI.
- T3a/T3b disease or suspected extracapsular extension warrants WB‑MRI escalation.
- Suspicion of metastatic disease should prompt WB‑MRI.
- Patient age and digital rectal examination findings should not influence GBCA administration or WB‑MRI escalation.

#### No consensus

- PSA level as a modifying factor for GBCA administration.
- PSA density thresholds, with divergent preferred cut‑offs (≥ 0.15–0.20 ng/mL/cm³).
- WB‑MRI escalation for isolated T3a/T3b disease without additional risk indicators.
- Use of WB‑MRI for unexplained pain in the absence of other concerning findings.

Qualitative feedback highlighted substantial variability in local practice, particularly in how centres interpret PI‑QUAL and apply PSA‑based criteria, reflecting heterogeneity in diagnostic pathways and reliance on biochemical versus imaging‑derived indicators.

Free‑text responses indicated that PSA, when considered at all, was generally treated as a contextual rather than deterministic modifier. Similarly, PSA density was not routinely incorporated in several institutions, and preferences for threshold selection varied widely.

A detailed summary of Round 2 agreement patterns is provided in Table 4.

**Table 4:** Delphi round 2 summary of agreement (*n* = 14 participants; consensus ≥ 70%)

Criteria	Consensus status	Agreement (%)	Agree (*n*)	Disagree (*n*)	Notes/qualitative insights
GBCA use for PI-RADS 3 lesions	Consensus	85.7	12	2	Majority support GBCA use for PI-RADS 3 lesions to improve diagnostic confidence; dissenters noted sufficient bpMRI performance.
Image quality (PI-QUAL threshold)	Consensus	71.4	10	4	Agreement that GBCA should be considered when PI-QUAL ≤ 3; not required at PI-QUAL 4 or PI-QUAL 5 (optimal image quality).
PSA level (GBCA decision)	No consensus	50.0	7	7	Mixed responses; several indicated no fixed PSA threshold, while others suggested ≥ 4 or ≥ 20 ng/mL as triggers.
PSA density (GBCA decision)	No consensus	57.1	8	6	No consensus around PSA density ≥ 0.15–0.20 ng/mL/cm³ prompting GBCA consideration; nearly half felt PSA density not practical or relevant.
Patient age (GBCA decision)	Consensus	85.7	12	2	Age alone should not influence the decision to use GBCA.
Non-use of DRE findings (GBCA decision)	Consensus	92.9	13	1	DRE findings should not influence GBCA use; viewed as outdated per GIRFT guidance.
WB-MRI participation/applicability	Consensus	100	14	0	All respondents completed WB-MRI section and confirmed relevance.
WB-MRI trigger: PSA ≥ 20 ng/mL	Consensus	85.7	12	2	PSA ≥ 20 ng/mL widely accepted as threshold for WB-MRI consideration.
WB-MRI trigger: T3a/T3b or ECE	Consensus	92.9	13	1	Strong consensus to perform WB-MRI in locally advanced disease (T3a/T3b or ECE/SVI).
WB-MRI trigger: suspicion of metastases	Consensus	92.9	13	1	WB-MRI recommended for radiologic or clinical suspicion of metastases; one respondent cautioned against overuse for nonspecific back pain.
Do not use age as a WB-MRI trigger	Consensus	85.7	12	2	Age should not influence WB-MRI referral.
Radiographer training outline	Consensus	100	14	0	All agreed training content appropriate; some suggested inclusion of quizzes or certification.

### Round 3 results

Eleven participants completed Round 3.

#### GBCA administration

Consensus was achieved for:

- PI-QUAL 1–2: administer GBCA or consider repeat scan (90.9%)
- PI-QUAL 3: administer GBCA (90.9%)
- PI-QUAL 4–5: do not administer GBCA (72.7%)

**Fig. 3:**
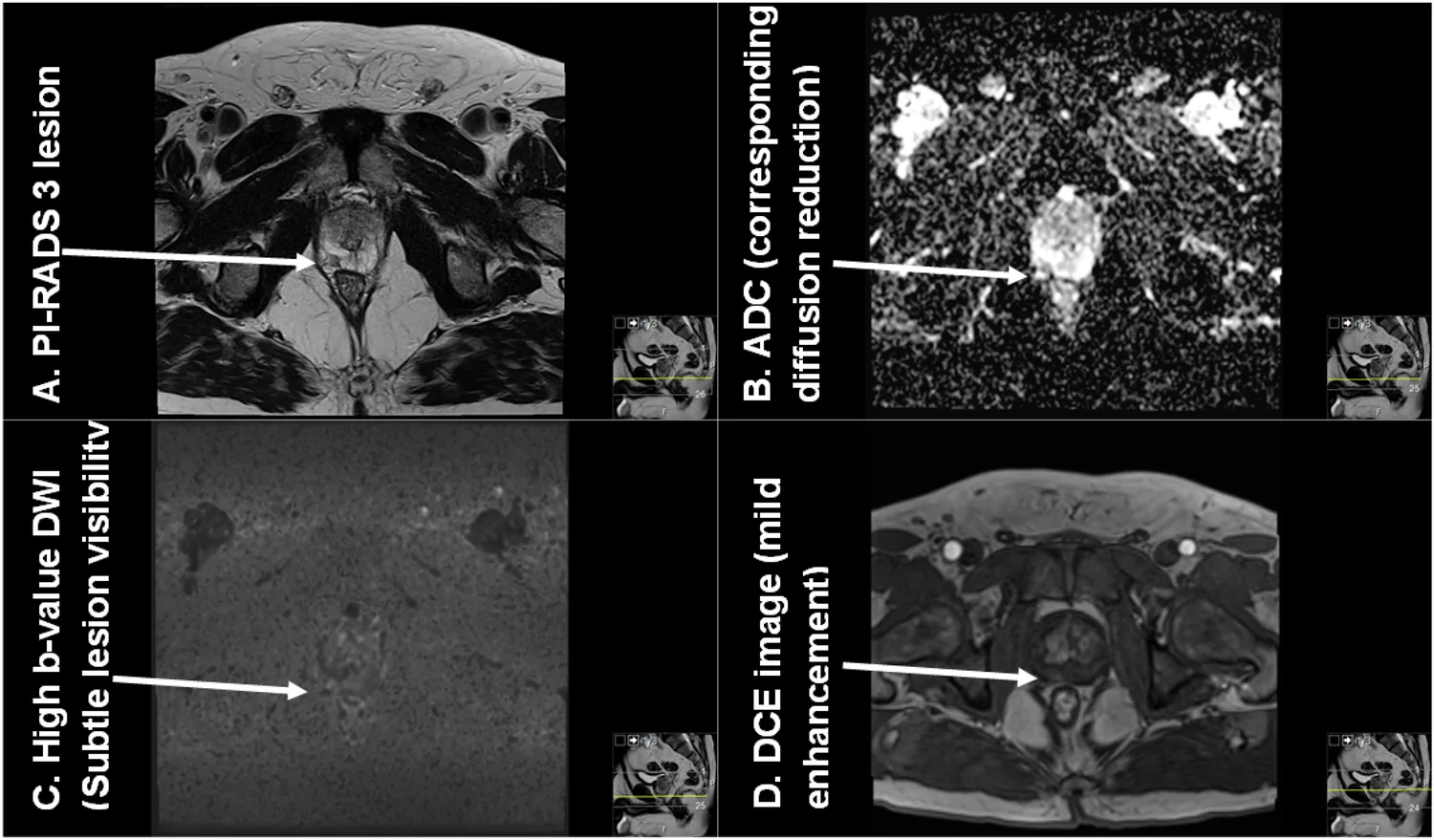
Representative PI-RADS 3 lesion in the peripheral zone. **A** Axial T2-weighted image demonstrates a focal hypointense lesion (arrow). **B** ADC map shows a corresponding low signal. **C** High b-value DWI demonstrates mild hyperintensity. **D** Dynamic contrast-enhanced imaging demonstrates mild post-contrast enhancement. This represents a typical equivocal lesion where selective GBCA administration may improve diagnostic confidence

**Fig. 4:**
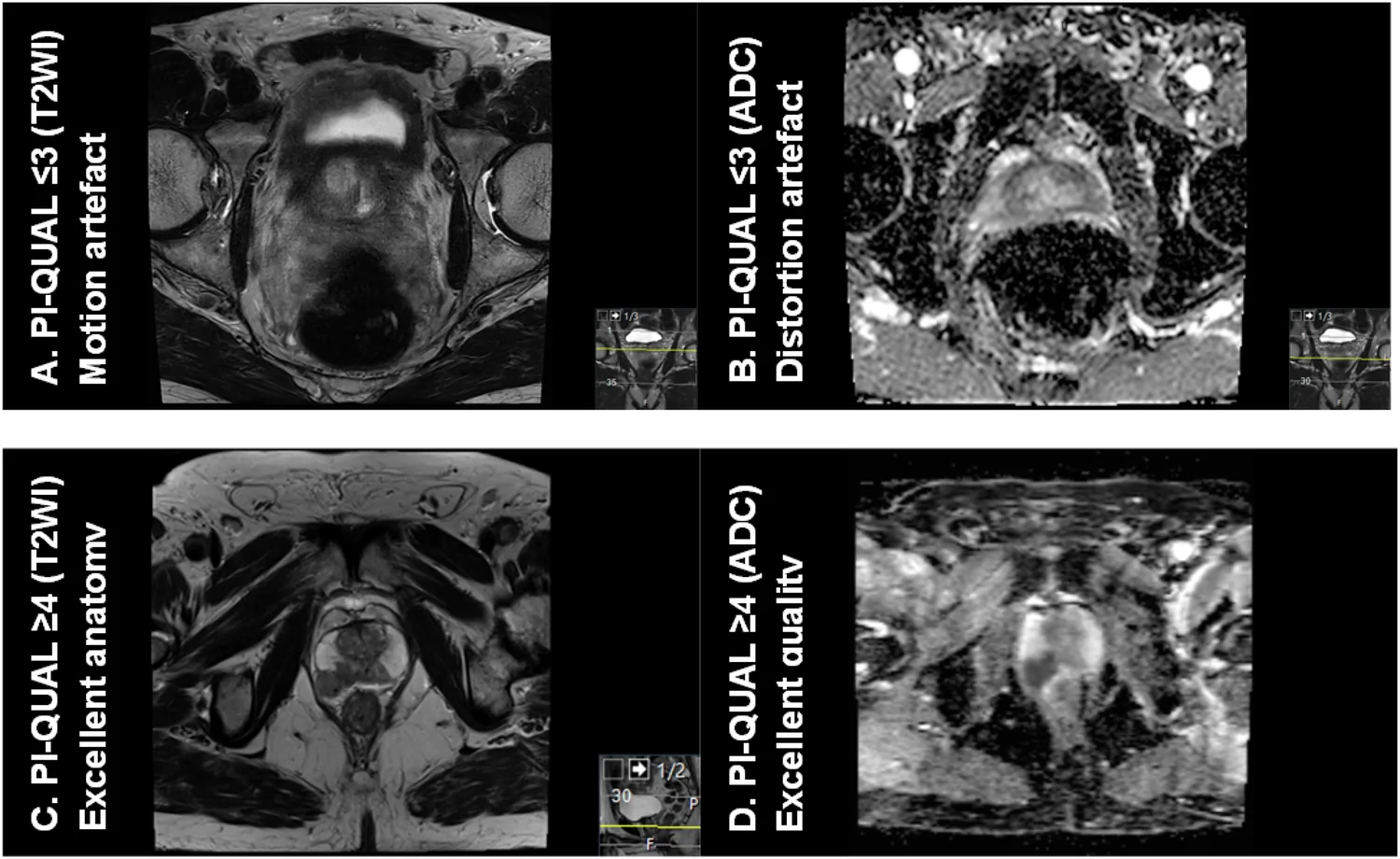
Comparison of image quality using PI-QUAL assessment. **A**, **B** PI-QUAL ≤ 3 examination demonstrating motion and susceptibility artefacts limiting lesion assessment. **C**, **D** PI-QUAL ≥ 4 examination demonstrating optimal image quality. Consensus supported selective GBCA administration or repeat imaging in lower-quality examination

Representative examples of findings supporting selective GBCA administration are shown in Fig. 3 and Fig. 4.

No consensus was reached for PSA density thresholds. Responses remained dispersed without consistent clustering.

#### WB-MRI escalation

Consensus was achieved for:

- T3a disease with PSA ≥ 15 ng/mL (90.9%)
- Combined clinical and radiological suspicion of metastasis (90.9%)
- Isolated T3a disease (72.7%)
- Unexplained persistent, localised pain (72.7%)

**Fig. 5:**
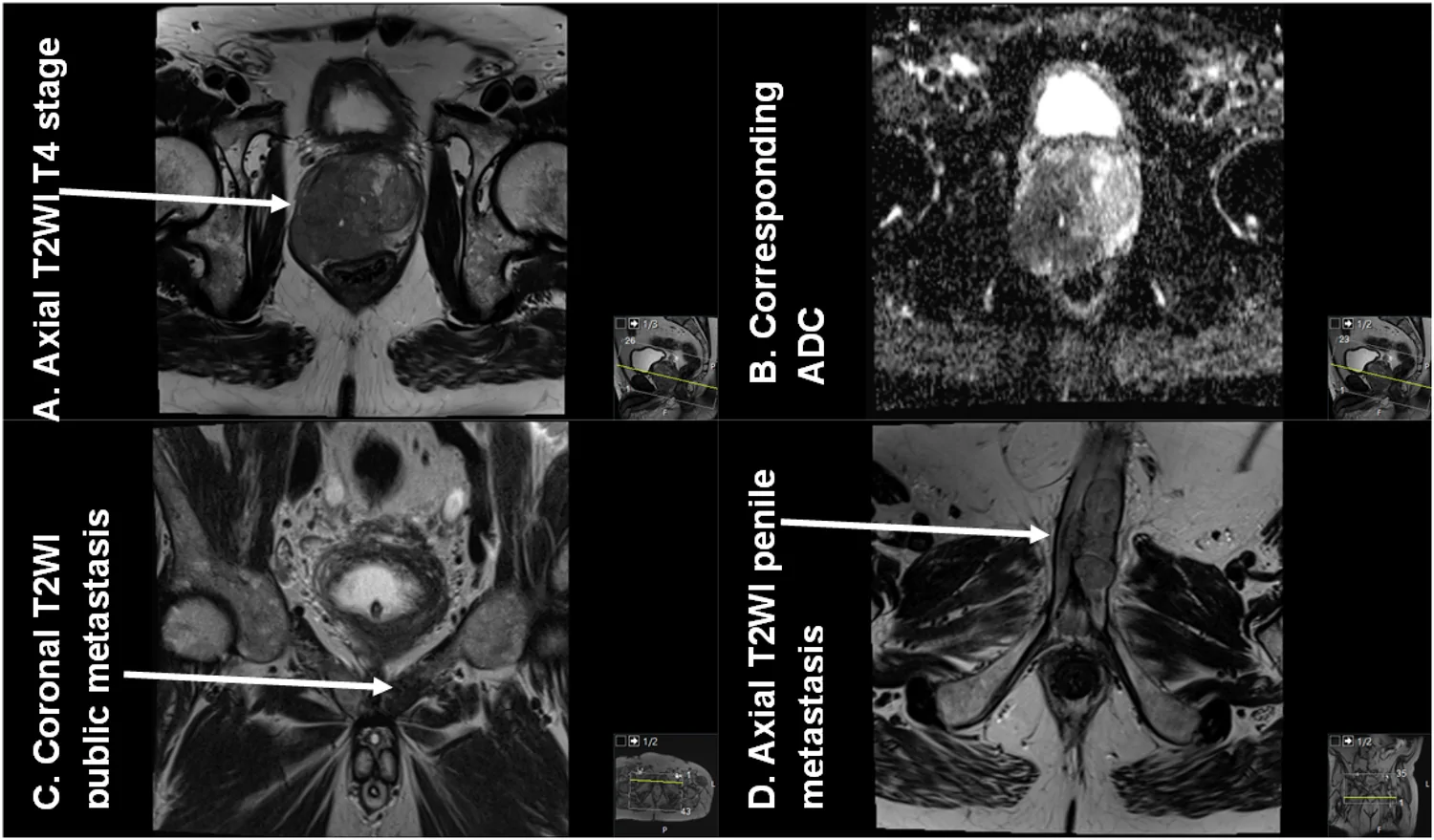
Examples of locally advanced prostate cancer. **A**, **B** T4 disease demonstrating extracapsular extension. **C**, **D** Metastasis to the pubic bone and penis. These features achieved a strong consensus as indications for WB-MRI escalation

Representative examples of locally advanced disease associated with escalation to WB-MRI are shown in Fig. 5.

Free‑text responses indicated that decisions regarding WB‑MRI escalation were often influenced by institutional MRI capacity, while GBCA‑related thresholds depended largely on radiologist familiarity with PI‑QUAL scoring.

A detailed summary is provided in Table 5.

**Table 5:** Delphi round 3 summary of agreement (*n* = 11 participants; consensus ≥ 70%)

Criteria	Consensus status	Agreement (%)	Agree (*n*)	Disagree (*n*)	Notes/qualitative insights
GBCA administration					
PI-QUAL 1–2 (suboptimal)—administer GBCA	Consensus	90.9	10	1	Strong consensus for repeat scan/GBCA use in poor-quality scans.
PI-QUAL 3 (borderline)—administer GBCA	Consensus	90.9	10	1	Broad support for GBCA use to improve diagnostic confidence.
PI-QUAL 4 (generally diagnostic)—do not administer GBCA	Consensus	72.7	8	3	GBCA not recommend when scan quality is sufficient.
PI-QUAL 5 (optimal)—do not administer GBCA	Consensus	72.7	8	3	GBCA unnecessary for optimal-quality scans.
PSA density ≥ 0.15 ng/mL/cm³—administer GBCA	No consensus	54.5	6	5	Near-equal split; uncertain role as modifying factor.
PSA density only should not influence GBCA	No consensus	45.5	5	6	Indicates lack of convergence on PSA density criteria.
WB-MRI escalation					
T3a (ECE) + PSA ≥ 15 ng/mL—escalate	Consensus	90.9	10	1	Strong support for escalation; considered high-risk context.
T3a (ECE) alone—escalate	Consensus	72.7	8	3	Moderate agreement; scenario wording influenced responses.
Combined clinical & radiological suspicion of metastasis—escalate	Consensus	90.9	10	1	Clear consensus to escalate to WB-MRI when both indicators present.
Localised unexplained persistent pain—escalate	Consensus	72.7	8	3	Moderate support; reflects variable thresholds and local practice.

## Discussion

This Delphi study provides the first structured expert consensus defining clinical and imaging criteria to support on-table decision-making for GBCA administration and WB-MRI escalation during prostate MRI. Previous consensus initiatives have focused primarily on acquisition standards, reporting frameworks and quality assurance [23]. However, none have defined operational criteria to support decision-making on when GBCA administration or WB-MRI is required.

The Delphi process was used to generate consensus‑based criteria that experts consider critical to safe on‑table decision‑making. Importantly, these findings represent expert consensus rather than empirical validation of diagnostic performance or clinical outcomes. While Delphi methodology provides structured synthesis of specialist opinion, the proposed consensus-based criteria should be regarded as guidance to inform decision‑making rather than evidence of improved diagnostic accuracy, safety or patient outcomes. Although participant numbers declined across rounds, attrition analysis demonstrated broadly comparable characteristics between those who continued and those who withdrew, reducing concerns regarding systematic attrition bias.

Across rounds, the panel prioritised PI-RADS and PI-QUAL as the primary determinants of GBCA administration. Consensus supporting GBCA administration for PI-RADS 3 lesions reflect expert reliance on contrast enhancement in equivocal cases. This is consistent with the literature suggesting that GBCA administration may improve characterisation of PI-RADS 3 lesions and modestly increase cancer detection, although findings remain heterogeneous across studies [24, 25].

Strong consensus also supported GBCA administration in PI-QUAL 1–3 examinations. Reduced MRI image quality is associated with lower diagnostic performance, increased uncertainty in lesion characterisation and a greater likelihood of additional downstream investigations [26–28]. In this context, GBCA administration may improve lesion conspicuity and diagnostic confidence [4], particularly in examinations of suboptimal but still diagnostic quality (e.g., PI-QUAL 3). By contrast, PI-QUAL 1–2 examinations, reflecting non-diagnostic image quality, may warrant repeat imaging where clinically appropriate. The inter-reader reproducibility and external validation of PI-QUAL [29, 30] further support its role in structured decision-making frameworks. Conversely, fixed biochemical thresholds such as PSA and PSA density did not achieve stable consensus across rounds. Responses remained dispersed, reflecting heterogeneity in local diagnostic pathways and differing reliance on biochemical versus imaging criteria. This variability aligns with prior research showing inconsistent performance of PSA density across clinical contexts [31]. These findings support the panel’s view that biochemical parameters should be applied as contextual rather than deterministic factors when guiding selective GBCA administration and WB‑MRI escalation in prostate MRI.

Consensus for WB-MRI escalation was stronger and more stable. High-risk features, including locally advanced disease (T3a/T3b) and combined clinical or radiological suspicion of metastases, were consistently endorsed and align with established high-risk stratification criteria [32]. Consensus for PSA ≥ 20 ng/mL and T3a disease with PSA ≥ 15 ng/mL aligns with national and international metastatic screening recommendations [26, 33]. These findings suggest greater uniformity in staging escalation decisions than in contrast-administration practices.

These consensus-based criteria establish a structured foundation for future research into workflow decision-support approaches; however, their operationalisation as a clinical decision-support system requires separate development and validation. As the panel also evaluated a radiographer training package, any future clinical decision-support system informed by these consensus-based criteria would need to be accompanied by structured training to ensure consistent and safe application.

## Limitations

Several limitations merit consideration. First, the findings reflect expert consensus rather than prospective validation and therefore represent informed opinion rather than outcome-based evidence. Prospective evaluation is required to determine diagnostic accuracy, safety, workflow efficiency and impact on patient outcomes.

Second, although attrition occurred across Delphi rounds, comparison of baseline characteristics between continuers and non-responders did not demonstrate a consistent or systematic difference in professional seniority, expertise, experience, reporting frequency or geographic distribution. While attrition cannot be entirely excluded as a potential source of bias and may introduce subtle self-selection effects, the available data do not suggest that the final consensus reflects a materially different subgroup of the original expert panel. In the final round, the reduced panel size (*n* = 11) meant that the predefined 70% consensus threshold corresponded to 8 of 11 participants, such that individual responses could influence borderline consensus classifications.

Third, the panel was UK-based, which may limit generalisability to healthcare systems with different imaging access, workforce models or staging pathways.

Finally, variability in PI‑QUAL interpretation across centres may influence the reproducibility of image‑quality–based decision rules, despite encouraging reliability data in the literature. The absence of stable consensus around PSA and PSA density thresholds further reflects variability in their clinical application across practice settings. Accordingly, these factors should be considered when interpreting the robustness and generalisability of the reported consensus.

## Conclusion

This Delphi consensus established structured MRI and clinical criteria to guide selective GBCA administration and WB‑MRI escalation in prostate MRI. While these criteria offer a coherent expert‑derived basis for on‑table decision‑making, prospective validation is required to determine their diagnostic performance, operational feasibility and impact on patient outcomes before clinical implementation.

## Supplementary information


ELECTRONIC SUPPLEMENTARY MATERIAL


## Data Availability

The datasets generated and analysed during this Delphi study are stored in Microsoft Excel format on a secure NHS Trust laptop. While the published analyses are based on anonymised data, participant email addresses were collected during the first survey round for study administration purposes. As such, the full datasets are not publicly available. The minimal anonymised dataset required to interpret and replicate the findings is available from the corresponding author on reasonable request, subject to institutional data governance approvals.

## Abbreviations

bpMRI: Biparametric magnetic resonance imaging
BSUR: British Society of Urogenital Radiology
CREDES: Conducting and Reporting of Delphi Studies
DRE: Digital rectal examination
ECE: Extracapsular extension
GBCA: Gadolinium-based contrast agent
mpMRI: Multiparametric magnetic resonance imaging
NHS: National Health Service
PCa: Prostate cancer
PI-QUAL: Prostate imaging quality
PI-RADS: Prostate Imaging-Reporting and Data System
PSA: Prostate-specific antigen
PSMA-PET: Prostate-specific membrane antigen positron emission tomography
WB-MRI: Whole-body magnetic resonance imaging
