# Supplementary material for: Delphi consensus of specialist radiologists on imaging criteria for gadolinium contrast use and whole-body MRI in prostate cancer
Source: Insights Imaging. 2026 Sep 2;17:216. doi: 10.1186/s13244-026-02388-0 (PMC13534386; doi:10.1186/s13244-026-02388-0)
Supplement: Supplementary file 1 [file 13244_2026_2388_MOESM1_ESM.pdf]

Delphi consensus of specialist radiologists on imaging criteria for gadolinium contrast use and whole-body MRI in prostate cancer

ELECTRONIC SUPPLEMENTARY MATERIAL

Insights Imaging (2026) Onwuharine EN, Clark AJ, Dimmock M, Hill J et al.

A Delphi survey with radiologists to determine features used to make prostate MRI decisions

"Accelerate my diagnosis by avoiding unpleasant bone or CT scans and eliminate the need for injected MRI contrast agents—helping me get the right treatment faster" – Patient A.

Are you a **radiologist with expertise in prostate MRI**? We need your help to complete this NIHR-funded survey. Patients have told us they are frustrated with having to receive unnecessary contrast agent injections and follow-up hospital appointments. **PLEASE HELP US SOLVE THE PROBLEM.**

We are conducting a Delphi survey with expert radiologists to identify the key features in prostate MRI decision-making. This will support the development of a clinical decision support system (CDSS) to guide radiographers in making real-time decisions along the patient's pathway. In case you are not aware, a CDSS is software designed to help healthcare professionals make real-time decisions. We are particularly interested in how best to make choices on gadolinium-based contrast agent (GBCA) injection and progression to whole-body MRI (WB-MRI) during prostate scans.

We will ask a few questions about your demographics and key clinical, laboratory, and imaging factors you consider for administering GBCA or upgrading a scan to WB-MRI. The survey includes three rounds that will take approximately 5 minutes each.

This survey is part of a multi-phase, mixed-methods study to explore a new prostate MRI pathway (NEWPRO**MRI**-PATHWAY) led by **Dr. Alex Clark (Consultant Urogenital Radiologist)** at University Hospitals of North Midlands (UHNM), and Professor Jonathan Hill and Dr. Matthew Dimmock at Keele University. The project is being completed by the **NIHR funded** PhD candidate Eric Onwuharine, also from UHNM. Project

Survey questions for participating consultant radiologists

FIRST ROUND

Consenting

1. I confirm that I am a radiologist with experience in prostate MRI reporting. I have read the participant information sheet and agree to take part in this research (Please tick as applicable).

   - ◯ No
   - ◯ Yes

Consenting(Copy)

2. I confirm that I am a radiologist with experience in prostate MRI reporting. I have read the participant information sheet and agree to take part in this research (Please tick as applicable).

- ◯ No
- ◯ Yes

Clinical decisions for Gadolinium Based Contrast Agent (GBCA).

3. In your experience, what are the key factors to consider when determining the need for gadolinium-based contrast agent (GBCA) injection in prostate MRI? Please list these factors and provide any reasoning where applicable. For example, would you use GBCA for borderline lesions, and why or why not?

4. What key features do you believe should be included in a Clinical Decision Support System (CDSS) to help radiographers determine when a GBCA injection is necessary for prostate MRI? For example, should factors like the PI-RADS score or PSA level be considered?

Clinical decisions for Whole-Body MRI (WB-MRI).

5. What are the key factors to consider when determining the need to upgrade a prostate MRI to a whole-body MRI (WB-MRI)? Please list these factors and provide a brief explanation if needed. For example, high-risk cancer or suspected metastases.

6. What key features do you believe should be included in a CDSS to help radiographers decide when to upgrade a prostate MRI to a WB-MRI? For example, PSA levels or signs of extracapsular extension.

General Questions on GBCA and WB-MRI.

7. To what extent do you believe a CDSS could effectively assist radiographers in determining when a GBCA injection is necessary for prostate MRI?

- Extremely confident
- Somewhat confident
- Neutral
- Somewhat not confident
- Extremely not confident

8. In what ways do you think a CDSS could assist radiographers in deciding when to upgrade a prostate MRI to a WB-MRI? For example, by flagging patients who may be at high risk of metastases.

9. Are you aware of any CDSSs that radiographers use in any form of imaging to assist in decision-making in their practice?

- Yes
- No

10. Are you aware of any CDSSs that radiographers use to assist in deciding when a WB-MRI or a GBCA injection is required for prostate MRI scans?

- Yes
- No

11. If you answered yes, to either questions 8 or 9, can you provide examples of these CDSSs?

12. In your opinion, what are the major challenges in implementing a CDSS in the prostate MRI workflow, and how might these challenges be overcome? For example, workflow disruptions caused by increased workload.

Demographics

13. What is the highest level of academic qualification you have obtained?

- ◯ Medical Degree (M.D. or D.O.)
- ◯ Internship/First Year Residency (1 year)
- ◯ Residency (3-7 years, depending on specialty)
- ◯ Fellowship (Subspecialization) (1-3 years)
- ◯ Board Certification (After completing residency or fellowship)
- ◯ Consultant/Attending Physician (Fully qualified)
- ◯ Academic Titles (e.g., Ph.D., Professor)

14. What is your primary area(s) of expertise?

15. How many years of experience do you have in prostate MRI reporting?

- ◯ Less than 5 years
- ◯ 5-10 years
- ◯ 10-20 years
- ◯ More than 20 years

16. How often do you report prostate MRI scans?

- ◯ Daily
- ◯ Weekly
- ◯ Monthly
- ◯ Seasonal
- ◯ Yearly
- ◯ Never

17. In what part of the UK is your practice based?

- England
- Wales
- Scotland
- Northern Ireland

Consenting for future rounds of this survey

18. We would like to invite you to participate in future rounds. Being involved in these rounds will give you the opportunity to review and provide feedback on the collective responses from this survey.

◯ Yes

◯ No

19. If yes, do you consent to your email address being securely stored and used exclusively for distributing future survey rounds?

◯ Yes

◯ No

20. If yes, please enter your email address in the box below.

This content is neither created nor endorsed by Microsoft. The data you submit will be sent to the form owner.

Microsoft Forms

A Delphi survey with radiologists to determine features used to make prostate MRI decisions

Second Round

* Required

Participation Confirmation (Optional)

1. Please confirm your continued participation in this Delphi process *

- ◯ Yes
- ◯ No

Gadolinium-Based Contrast Agent (GBCA) Use

2. *The following clinical variables were identified in Round 1 as influencing the decision to use gadolinium-based contrast agents (GBCA) in prostate MRI:*

- PI-RADS 3 lesions (to support diagnosis)
- Poor-quality DWI sequences
- Elevated PSA/PSA density with suspicious lesions
- Older patient age (e.g. ≥65 years)
- Abnormal DRE (e.g. nodules, asymmetry, firmness)

Do you believe this list adequately reflects the key variables used in clinical decision-making?
*

○ Yes

○ No

3. If you selected "No", please list any additional factors you believe should be included and explain why:

Selection of Thresholds for GBCA Administration in Prostate MRI

For each clinical factor below, please select the threshold you believe is most appropriate for guiding the decision to administer gadolinium-based contrast agents (GBCA) during prostate MRI. You may also suggest an alternative threshold if none of the options reflect your clinical judgment.

4. Image quality: At what PI-QUAL score/threshold should GBCA be considered to improve diagnostic confidence? **PI-QUAL** is a score from 1 (poor) to 5 (excellent) that rates the quality of prostate MRI scans for reliable cancer detection. *

- ◯ PI-QUAL 1–2: GBCA should be used because image quality is poor or non-diagnostic on bpMRI.
- ◯ PI-QUAL 3: GBCA should be considered to improve diagnostic confidence due to borderline image quality.
- ◯ PI-QUAL 4: GBCA may be used selectively; image quality is generally diagnostic but minor improvements could help.
- ◯ PI-QUAL 5: GBCA is generally not required as image quality is optimal on bpMRI.
- ◯ No specific threshold should be applied
- ◯ Other

5. If you selected ‘Other’, please specify:

6. PSA Level: At what PSA level should contrast-enhanced imaging be considered? *

- ◯ ≥4 ng/mL
- ◯ ≥10 ng/mL
- ◯ Use age-specific PSA thresholds
- ◯ No fixed PSA threshold
- ◯ Other

7. If you selected ‘Other’, please specify:

8. PSA Density: What PSA density threshold should prompt consideration of GBCA use? *

- ◯ ≥0.10 ng/mL/cm³
- ◯ ≥0.15 ng/mL/cm³
- ◯ ≥0.20 ng/mL/cm³
- ◯ PSA density should not influence GBCA decision
- ◯ Other

9. If you selected ‘Other’, please specify:

10. Patient Age: At what age should increased risk prompt routine consideration of GBCA-enhanced imaging? *

- ◯ ≥60 years
- ◯ ≥65 years
- ◯ ≥70 years
- ◯ ≥80 years
- ◯ Age should not be a factor
- ◯ Other

11. If you selected ‘Other’, please specify:

12. Digital Rectal Examination (DRE) Findings: Which of the following DRE findings should influence the decision to administer GBCA? Please select all options that apply. *

- [ ] Palpable nodule
- [ ] Asymmetry
- [ ] Induration
- [ ] Firm gland
- [ ] Fixed or hard gland
- [ ] Any other abnormal DRE finding
- [ ] DRE findings should NOT influence GBCA use
- [ ] Other

13. If you selected 'Other', please specify:

Escalation to Whole-Body MRI (WB-MRI) during Prostate MRI Evaluation

14. *Based on the factors identified in Round 1 as influencing the decision to upgrade to whole-body MRI (WB-MRI), including:*

    - *PSA≥20 ng/mL.*
    - *T3a/T3b lesions and extracapsular extension.*
    - *Suspicion of metastases (e.g., bony lesions or relevant pain symptoms e.g. back or pubic pain).*

    Do you believe this list adequately reflects the key clinical factors relevant to the decision to upgrade to WB-MRI? *

    ◯ Yes

    ◯ No

15. If you selected "No", please list any additional factors you believe should be included and explain why:

Selection of threshold and factors for escalation to WB-MRI following prostate MRI

For each clinical factor below, please select the threshold you believe is most appropriate for escalating to WB-MRI during prostate MRI. You may also suggest an alternative threshold if none of the options reflect your clinical judgment.

16. PSA level *

- ○ PSA ≥ 20 ng/mL
- ○ PSA ≥ 15 ng/mL
- ○ PSA ≥ 10 ng/mL
- ○ PSA ≥ 5 ng/mL
- ○ Other

17. If you selected ‘Other’, please specify:

[ ]

18. T3a/T3b lesions and extracapsular extension (ECE). Please select all options that apply. *

- ☐ Escalate if T3a lesion (ECE) identified
- ☐ Escalate if T3a with PSA ≥ 15 ng/mL
- ☐ Escalate if T3b (seminal vesicle invasion)
- ☐ Other

19. If you selected ‘Other’, please specify:

[ ]

20. Suspicion of metastases (e.g., bony lesions or relevant pain symptoms such as back or pubic pain). Please select all options that apply. Escalation Thresholds:
*

- [ ] Localised unexplained persistent pain suggestive of metastatic disease (e.g., low back or pubic pain).
- [ ] Imaging-identified bone lesions of uncertain significance, with or without unexplained pain.
- [ ] Radiological suspicion of metastases (e.g., bone lesions with concerning features), with or without unexplained pain.
- [ ] Combined clinical and radiological suspicion (e.g., concerning imaging features and relevant pain symptoms)
- [ ] Other

21. If you selected 'Other', please specify:

22. Patient Age: At what age should increased risk prompt routine consideration of WB-MRI? *

- ◯ ≥60 years
- ◯ ≥65 years
- ◯ ≥70 years
- ◯ ≥80 years
- ◯ Age should not be a factor
- ◯ Other

23. If you selected 'Other', please specify:

Proposed Training for Radiographers

We are developing a training package to support radiographers in using a clinical decision support system (CDSS) for prostate MRI. The training will focus on improving confidence and competence in decision-making around gadolinium-based contrast agent (GBCA) use and whole-body MRI (WB-MRI). The training is likely to be delivered through an online portal and will include:

- Webinars
- Case reviews
- Tutorials
- Short videos
- Virtual or augmented simulated reporting
- Quizzes
- Certification upon completion

It will also cover key topics such as relevant prostate anatomy and the measurement principles that underpin CDSS threshold decisions, enabling radiographers to apply the tool effectively in clinical practice.

24. **Module 1 – Foundations**

- Mode: Webinar and self-study
- Topics: Prostate anatomy, MRI basics, PSA, measurement principles
- Outcome: Understand anatomy, clinical relevance, and basic measurements

Module 2 – Case-Based Learning

- Mode: Interactive case reviews
- Topics: 4–6 anonymised MRI cases, PSA density, group discussion
- Outcome: Apply knowledge, identify pathology, collaborate

Module 3 – Practical Workshop

- Mode: Hands-on with expert tutor
- Outcome: Practice accurate measurement, build confidence
- Topics: MRI tools, segmentation, live demo, feedback

Module 4 – Reporting & Reflection

- Mode: Simulated reporting
- Topics: Write structured reports, peer/tutor feedback, reflection
- Outcome: Consolidate skills, improve reporting, plan development

25. Do you believe this training outline adequately reflects the topics and content relevant for radiographers to use the prostrate MRI clinical decision support system? *

- ◯ Yes
- ◯ No

26. If you selected "No", please list any additional topics you believe should be included and explain why:

Thank you for completing Round 2 of this Delphi survey.
Your input is greatly appreciated and will contribute to the development of a CDSS for prostate MRI, particularly regarding GBCA use and WB-MRI. Responses will now be analysed, and a summary of key findings will be shared with participants in due course. You may be contacted if a further round is required. *Eric N. Onwuharine* - On behalf of the NEWPROMRI-PATHWAY Study Team.

This content is neither created nor endorsed by Microsoft. The data you submit will be sent to the form owner.

Microsoft Forms

A Delphi survey with radiologists to determine features used to make prostate MRI decision

THIRD ROUND

Thank you for participating in Round 3, the final round of this Delphi consensus study. In Round 2, consensus was achieved for several variables; however, some areas remained unresolved. This round focuses **only on those unresolved items**. Please answer each question based on your expert judgment. Consensus is predefined as ≥75% agreement.

* Required

Section 1: GBCA Administration

1. **PI-QUAL** is a score from 1 (poor) to 5 (excellent) that rates the quality of prostate MRI scans for reliable cancer detection.

   For suboptimal scans (PI-QUAL 1–2), should GBCA be administered?

   Round 2 feedback:

   - PI-QUAL 1–2: 14.3% Yes

   *

   ◯ Yes, recommend GBCA

   ◯ No, do not recommend GBCA

2. For suboptimal scans (PI-QUAL 3), should GBCA be administered?

   Round 2 feedback:

   - PI-QUAL 3: 21.4% Yes

   *

   ◯ Yes, recommend GBCA

   ◯ No, do not recommend GBCA

3. For generally diagnostic scans (PI-QUAL 4), should GBCA be administered?

Round 2 feedback:

- PI-QUAL 4 28.6% Yes

*

- ◯ Yes, recommend GBCA
- ◯ No, do not recommend GBCA

4. For generally optimal scans (PI-QUAL 5), should GBCA be administered?

Round 2 feedback:

- PI-QUAL 5 35.7% Yes

*

- ◯ Yes, recommend GBCA
- ◯ No, do not recommend GBCA

5. PSA as a Determinant for GBCA: Which option best reflects your recommendation? *(Single choice)*

Round 2 feedback:

- 50% suggested no fixed threshold
- 35.7% age-specific
- 7.1% ≥4 ng/mL
- 7.1% unclear

*

- ◯ Do not use PSA alone to guide GBCA
- ◯ Use age-specific PSA thresholds
- ◯ Apply a fixed PSA threshold
- ◯ Other

6. If you selected 'Apply a fixed PSA threshold or Other' for Question 4, please specify.

7. PSA Density: Which approach should be used? *(Single choice)*

Round 2 feedback:

- 42.9% ≥0.15 ng/mL/cm^3,
- 14.3% ≥0.20 ng/mL/cm^3,
- 35.7% should not influence,
- 7.1% unclear.

*

- ◯ PSA density should not influence GBCA decisions
- ◯ PSA density ≥0.15 ng/mL/cm^3
- ◯ PSA density ≥0.20 ng/mL/cm^3
- ◯ Other

8. If you selected 'Other' for Question 6, please specify.

Section 2: WB-MRI Escalation

9. T3a Lesions; please indicate for each scenario in questions 9 and 10:

T3a with PSA ≥15 ng/mL

Round 2 feedback:

- 57.1% Yes

*

◯ Escalate

◯ Do not escalate

10. T3a (ECE)

Round 2 feedback:

- 50.0% Yes

*

◯ Escalate

◯ Do not escalate

11. Metastatic Suspicion; please indicate for each scenario in questions 11 and 12:

Combined clinical and radiological suspicion.

Round 2 feedback:

- 57.1% Yes

*

◯ Escalate to WB-MRI

◯ Do not escalate

12. Localised unexplained persistent pain (e.g., lumbosacral spine)

Round 2 feedback:

- 50.0% Yes

*

◯ Escalate to WB-MRI

◯ Do not escalate

Optional Comments

13. Please provide any comments or clarifications regarding GBCA administration or WB-MRI escalation rules.

14. **Thank you for your participation in this Delphi survey. Your valuable input is greatly appreciated and will help shape the development of a CDSS for prostate MRI.** *Eric N. Onwuharine* - On behalf of the NEWPROMRI-PATHWAY Study Team.

This content is neither created nor endorsed by Microsoft. The data you submit will be sent to the form owner.

Microsoft Forms

Supplementary File 2

CREDES Checklist Compliance Summary

CREDES Domain	Item	How Addressed in This Study
1. Rationale	Justification for Delphi	A modified e-Delphi method was used to synthesise structured expert consensus on selective GBCA use and WB-MRI escalation, appropriate due to heterogeneity in published evidence and variability in clinical practice.
2. Planning and Design	Design description	Three-round modified e-Delphi with Round-1 items derived from semi-structured interviews and a systematic review; all rounds electronic with structured quantitative and qualitative feedback.
	Consensus definition	Consensus was predefined as ≥70% agreement on Likert-scaled items. Items meeting threshold were accepted; others modified or excluded based on feedback.
3. Study Conduct	Informational input	Round-1 content derived from qualitative interviews with prostate MRI radiologists and a systematic

		review of imaging and clinical decision variables.
	Expert panel selection	Panel comprised consultant radiologists with ≥1 year independent prostate MRI experience, ≥5 weekly scans, and familiarity with PI-RADS and PI-QUAL; recruited via BSUR.
	Panel size and attrition	• Round-1 (R1): 21<br>• Round-2 (R2): 14<br>• Round-3 (R3): 11.<br>Attrition analysed between R1–R2 with no systematic bias identified.
	Anonymity and bias prevention	Responses anonymised; participants blinded to one another; controlled feedback prevented dominance and conformity bias.
	Feedback process	Participants received percentage agreement, anonymised comments, and their own previous responses between rounds.
	Data analysis	Quantitative agreement summarised descriptively; qualitative free-text analysed thematically with inter-rater reliability ($\kappa = 0.755$).

	External validation	No external validation conducted; findings presented as expert guidance requiring future evaluation.
4. Reporting	Purpose	To generate structured expert consensus on MRI and clinical criteria informing selective GBCA use and WB-MRI escalation.
	Methods transparency	Methods detailed in manuscript, including preparatory work, Delphi rounds, consensus threshold, feedback, and analysis.
	Consensus attainment	Consensus (≥70%) guided item retention, modification, or exclusion across rounds.
	Results reporting	Round-specific agreement levels, dissent patterns, and qualitative insights reported to show consensus development.
	Limitations	Limitations include use of expert opinion, UK-based sample, attrition, variability in PI-QUAL interpretation, and lack of PSA consensus.
	Conclusions	Conclusions reflect expert-derived criteria requiring prospective

		validation prior to clinical implementation.
	Dissemination	Findings are intended to support radiographer and radiologist workflow standardisation, improve consistency in on-table decision-making, and inform future validation and implementation studies.
